# Transmit Radiant Individuality to Offspring (TRIO) study: investigating intergenerational transmission effects on brain development

**DOI:** 10.3389/fpsyt.2023.1150973

**Published:** 2023-09-28

**Authors:** Izumi Matsudaira, Ryo Yamaguchi, Yasuyuki Taki

**Affiliations:** ^1^Frontier Research Institute for Interdisciplinary Sciences, Tohoku University, Sendai, Japan; ^2^Smart-Aging Research Center, Tohoku University, Sendai, Japan; ^3^Japan Society for the Promotion of Science, Tokyo, Japan; ^4^Department of Medical Sciences, Graduate School of Medicine, Tohoku University, Sendai, Japan

**Keywords:** intergenerational transmission, parent-offspring trios, neuroimaging, development, personality

## Abstract

Intergenerational transmission is a crucial aspect of human development. Although prior studies have demonstrated the continuity of psychopathology and maladaptive upbringing environments between parents and offspring, the underlying neurobiological mechanisms remain unclear. We have begun a novel neuroimaging research project, the Transmit Radiant Individuality to Offspring (TRIO) study, which focuses on biological parent-offspring trios. The participants of the TRIO study were Japanese parent-offspring trios consisting of offspring aged 10–40 and their biological mother and father. Structural and functional brain images of all participants were acquired using magnetic resonance imaging (MRI). Saliva samples were collected for DNA analysis. We obtained psychosocial information, such as intelligence, mental health problems, personality traits, and experiences during the developmental period from each parent and offspring in the same manner as much as possible. By April 2023, we completed data acquisition from 174 trios consisting of fathers, mothers, and offspring. The target sample size was 310 trios. However, we plan to conduct genetic and epigenetic analyses, and the sample size is expected to be expanded further while developing this project into a multi-site collaborative study in the future. The TRIO study can challenge the elucidation of the mechanism of intergenerational transmission effects on human development by collecting diverse information from parents and offspring at the molecular, neural, and behavioral levels. Our study provides interdisciplinary insights into how individuals’ lives are involved in the construction of the lives of their descendants in the subsequent generation.

## 1. Introduction

Interdisciplinary research involving human neuroimaging has contributed to understanding individuality by exploring the associations between genes, the environment, and gene–environment interactions and brain structure or function. Several large cohort studies, such as the Adolescent Brain Cognitive Development^SM^ (ABCD study®) study^1^ (1), IMAGEN study^2^ (2), Philadelphia Neurodevelopmental Cohort (PNC)^3^ (3), Generation R study^4^ (4), and Chinese Imaging Genetics (CHIMGEN) study^5^ (5) have taken the lead of this research area. However, in many cases, these large-scale studies do not cover a crucial aspect of human development—intergenerational transmission.

Intergenerational transmission refers to offspring inheriting behaviors and characteristics from parents through genetic and non-genetic pathways (6). Numerous studies have reported on the intergenerational continuities in psychopathology (7–12). Recent large-sample studies have revealed that children with parents who have a history of psychiatric hospitalization are at 2–3 times higher risk of developing mental illness in adolescence compared with those whose parents do not (13). Children who have experienced a parental suicide attempt during early childhood are likely to exhibit suicide attempts in their adolescence (14). Thus, intergenerational transmission of psychopathology is likely one of the crucial topics in current psychiatry (12). Furthermore, children tend to experience social interactions that are similar to those of their parents. For instance, it has been reported that families with a parent who was abused in childhood are confronted with a higher incidence of maltreatment than those with no abused parent (15, 16). Aside from maltreatment, variations in parenting behavior in a normative range, such as affection and harsh discipline, also seem to be transmitted intergenerationally (17–20). In addition, children whose fathers have experienced peer rejection in childhood are reportedly more likely to be victims of bullying themselves (21).

The experience of psychopathology or social interactions, as well as the effect of the experience could be inherited by offspring. Intergenerational transmission of trauma effect (intergenerational trauma) means that individuals’ adverse experiences, particularly in their childhood, affect their offspring (22). Adverse childhood experiences (ACEs) refer to exposure to threat and/or deprivation during childhood, including physical, psychological, or sexual abuse, and household dysfunction such as mental illness, substance abuse, criminal behavior, and violence between family members (23). Many recent studies have demonstrated the associations between maternal ACEs and offspring’s behavioral and neural phenotypes. For example, maternal ACEs are positively correlated with internalizing or externalizing symptoms and negatively correlated with resilience and self-affirmation in offspring (24, 25). However, it remains unclear what mechanisms are in place for parents and offspring to have the same experiences, or for the effect of parental life experiences to be passed on to offspring.

There are three notable concepts in the exploration of mechanisms of intergenerational transmission: intergenerational neuroimaging, epigenetic inheritance, and genetic nurture. First, intergenerational neuroimaging—a research field that uses brain images as endophenotypes to estimate the mechanisms of intergenerational transmission—is beginning to progress (26). Several pioneering studies have investigated the structural or functional brain similarities between parent–offspring dyads using magnetic resonance imaging (MRI) (27–40). These studies assumed that the stronger the correlation of brain characteristics between parents and offspring, the more similar their brains. It has been confirmed that parent–offspring dyads show greater similarity in brain characteristics than randomly selected adult–child pairs (35, 38, 39). Parent–offspring similarities have been reported in various characteristics, such as in gray matter volume (28, 35, 40), cortical thickness (29), surface area (39, 40), sulcal morphology (38, 39), fractional anisotropy (34), white matter microstructure (31, 32), resting-state functional connectivity (35–37), gumma-amino butyric acid and glutamate ratio (33), and task-evoked neural activation during reward processing (30). It seems that parent-offspring neural similarities do not reflect the offspring’s inheritance of particular brain characteristics from the parent but rather reflect the similarities in the process of brain development. Therefore, while it is difficult to clarify the molecular basis of intergenerational transmission through intergenerational neuroimaging, putative mechanisms can be deepened by understanding the brain characteristics that are similar between parent and offspring, genetic or environmental factors that are associated with the similarity, genetic or environmental factors associated with the development of brain characteristics that parent-offspring similarities are detected, including how the neural susceptibility for those factors are determined. Additionally, the association between parental ACEs and offspring’s brain development has been investigated to show the putative mechanisms of intergenerational trauma. For example, significant associations between maternal experiences of childhood maltreatment and infants’ total brain volume (41), total intracranial volume (42), amygdala volume (43), and functional connectivity in the frontoamygdala circuits (44) have also been reported. In one study, children whose mothers experienced a great earthquake in Turkey demonstrated smaller gray matter volumes in the hippocampus and amygdala (45). Second, epigenetic inheritance has been suggested to underlie the intergenerational transmission of trauma effect. Epigenetic inheritance means that life experiences and environmental exposures cause stable changes in non-DNA molecules in germ cells and alter gene expression patterns after embryonic development, thereby affecting the offspring’s phenotype (46). DNA methylation due to the effects of traumatic experiences has been observed in the same genes in both parents who had experienced it themselves and their children who had not (47–49). However, details of the association between epigenetic inheritance and intergenerational transmission of trauma effects have not been elucidated clearly. Third, the genetic nurture effect refers to the phenomenon that parental nature (i.e., parental genotype) affects offspring’s outcome through shaping the nurture (environments that parents provide to their offspring) (50). A prior study has robustly confirmed that parental polygenic risk scores of educational attainment calculated with non-transmitted alleles affect children’s educational outcomes (51). The mechanisms by which the genetic nurture effect is established include the effects of parental non-transmitted alleles on the prenatal environment, placenta, and postnatal environment, such as breast milk composition and nurturing behavior (46). Some researchers argue that clarification of what environmental factors related to parental non-transmitted alleles affect offspring’s educational outcomes will enable the development of interventions to break the chain of low educational attainment (51).

One of the reasons why the mechanisms of intergenerational transmission are unclear, despite the accumulation of previous studies as described above, is the lack of data on fathers. Although some studies examined brain similarities among all patterns of parent–offspring gender combinations (mother–daughter, mother–son, father–daughter, and father–son) (28, 40), most studies have focused on mother–offspring dyads (29, 30, 37–39), with some focusing on mother–daughter dyads (29, 30). Even in the context of intergenerational trauma, studies that focused on fathers are rare, but some interesting findings have been reported. For instance, paternal early-life stress was significantly correlated with neonates’ white matter microstructure in the corpus callosum (52). Furthermore, paternal ACEs are associated with offspring attention problems at 3 years of age, and this association is affected by the offspring’s blood DNA methylation in neonates (53). On the other hand, genetic studies have emphasized the significance of family-based designs like parent-offspring trios (54, 55). These previous studies have revealed gene–environment correlations (rGE) in the context of child development. Krapohl et al. found that offspring’s polygenic scores confer schizophrenia or educational attainment predicts the exposure to environmental factors such as paternal age at birth, maternal smoking during pregnancy, breastfeeding, parental smacking, household income, watching television, and maternal education level (54). Baldwin et al. have confirmed the genetic confounding effects in the association between ACEs and mental health, by clarifying that children with higher polygenic scores for mental health problems (attention-deficit hyperactivity disorder, depression, and schizophrenia) are more likely to be exposed to ACEs (55). However, both of these rGE studies had only offspring’s genotype data; therefore, parental genotype data is necessary to distinguish the observed rGE as passive (parental genotype associated with some environments are inherited by offspring) or evocative (offspring’s genotype evokes parental behavior). As suggested in previous studies, most offspring’s environments are parental phenotypes (54). Therefore, utilizing paternal, maternal, and offspring genotype and phenotype data is worthwhile for understanding the intergenerational effects of genetics and environments on human development.

In the field of neuroimaging, data from both parents and offspring are rarely available. Although birth cohorts and three-generational cohorts have contributed to a wealth of genomic information and physiological indices for parents and offspring, in many cases, brain images are obtained only from parents or offspring. For example, the Norwegian Mother and Child Cohort Study (MoBa)^6^ (56) and Avon Longitudinal Study of Parents and Children (ALSPAC)^7^ (57) have acquired brain MRIs from only a subset of children (58, 59). Additionally, although the Developing Human Connectome Project (dHCP)^8^ (60), which investigates typical and atypical brain development beginning from the fetal period, has collected maternal medical and obstetric histories, brain MRIs are only available for offspring. Three-generation cohorts such as the Tohoku Medical Megabank Project Birth and Three-Generation Cohort Study (TMM BirThree Cohort Study)^9^ (61) and LifeLines^10^ (62) have the possibility of collecting multi-generational brain images in the future. However, brain imaging has been performed only on some adults in the TMM BirThree Cohort Study and only on participants in a subproject (ImaLife) of LifeLines; thus, at least for now, multi-generational neuroimaging remains unavailable. In contrast, the population-neuroscience cohort studies of the Tokyo TEEN Cohort (pn-TTC)^11^ (63) and Generation R study (64) have obtained brain images of both parents and offspring. Most parental brain images in the pn-TTC were from the mother (35). For the Generation R study, it has not yet been reported whether both mothers and fathers completed MRI acquisition. Nonetheless, it seems that data on fathers are still lacking in studies on intergenerational transmission.

Consequently, for elucidating the mechanisms of intergenerational transmission, it is necessary to collect behavioral indices, brain images, environmental factors, and genetic/epigenetic information on at least two generations of parents and offspring. Hence, we started a novel neuroimaging research project—the Transmit Radiant Individuality to Offspring (TRIO) study—which is subject to biological parent-offspring trios comprising fathers, mothers, and offspring. This study aimed to elucidate the bio-psycho-social mechanism of intergenerational transmission using brain MRI as an endophenotype. We anticipate that the understanding of the role of parental genotype and/or life experiences in the development of personality and risk of mental illness in the subsequent generation would be deepened in this study. The findings of this study will contribute to the growth of effective interventions for individuals’ adaptive development. We hypothesized that: (1) maternal and paternal intergenerational transmissions are established by different mechanisms, and (2) the interaction between the paternal and maternal effects involves intergenerational transmission. The procedure for testing these hypotheses is presented using the effect of the parental experience of childhood maltreatment on offspring development as an example. Hypothesis (1) would be examined by comparing the result of the analyses using father-offspring dyads and mother-offspring dyads. For example, the paternal experience of childhood maltreatment and such experience of mothers may associate with neural and/or behavioral phenotype of offspring in a different way. Hypothesis (2) would be tested by analyzing whether the effect of the maternal experience of childhood maltreatment on offspring’s brain structure is moderated by the paternal childhood experience of being reared, and vice versa.

In this paper, we describe the acquiring data content, procedures of data collection, current progress, and future directions of our project.

## 2. Methods

### 2.1. Participants

The participants of the TRIO study were parent-offspring trios consisting of three members: a male or female offspring aged 10 (a 5th-grade elementary school student in Japan) to 40 and their biological mother and father. Most previous studies aiming to elucidate the mechanisms of intergenerational transmission have focused on offspring from infancy to adolescence. Therefore, it is unclear whether the effects of intergenerational transmission are observed at any age, or whether they are limited to certain ages (6). To the best of our knowledge, the maximum age of offspring is late 20s to 30 years in previous studies of parent-offspring brain similarity and the association of parental ACEs and offspring’s brain (40, 65). Thus, we set the upper age limit at 40 years, which is not included in previous studies and is classified as young adulthood in the developmental stage (66). In studies exploring early childhood, children’s personality and mental health problems are often assessed by parents. However, age-related differences in certain traits may prevent the detection of intergenerational transmission effects, for example, adult and child aggression appear as different behaviors. To mitigate this limitation, it is considered necessary to unify methods of data collection between parents and offspring. Thus, we set the lower age limit at 10 years, the age at which a person is considered to be able to answer the self-administered questionnaires. No maximum or minimum age limit was set for parents. To align the genetic backgrounds of the participants, it was required for all relatives within the third degree of kinship to be Japanese. Participants were required to have no history of cerebrovascular disease, brain tumor, intracranial disease, degenerative brain disease, epilepsy, serious heart disease, serious brain injury with impaired consciousness, no tendency of claustrophobia and nyctophobia, no metals in the body such as a cardiac pacemaker, and no possibility of current pregnancy. These conditions were verified at the time of participation, and if any member of the trio met the exclusion criteria, their participation was declined. If a participant was found to meet the exclusion criteria during participation in the study, only assessments that guaranteed safety were performed.

### 2.2. Incentives for participants

Each participant received a gift voucher worth 5,000 JPY. The parents were also given a report on brain health of themselves based on hippocampal volume using BrainSuite, developed by CogSmart, Inc. Offspring were given a printed copy of the T1-weighted brain image and a score report from the Wechsler Adult Intelligence Scale Fourth Edition (WAIS-IV) (67) or Wechsler Intelligence Scale for Children Fourth Edition (WISC-IV) (68).

### 2.3. Recruitment of participants

Advertisements for recruiting participants were published in a local magazine on town information. Additionally, flyers and posters were displayed at universities, high schools, vocational schools, government offices, public facilities, and stores in Sendai. All advertisements included information on the eligibility criteria, an overview of the inspections to be conducted, and the time required along with dates and rewards. A QR code on the advertisement could be scanned to access a website with details of the study. This website allowed applicants to access an application form created using Google Forms, in which applicants and their family members input their name, age, gender, role in the family (father, mother, or child), phone number, e-mail address, preferred date of participation, and confirmation that they do not meet the exclusion criteria. After reviewing the applicants’ input data, the staff coordinated the date and time of participation by e-mail or phone. If the number of applicants exceeded the targeted number of trios, a waiting list was created. Applicants in this list were requested to participate as soon as additional survey dates were added.

### 2.4. Experimental procedure

#### 2.4.1. Overview

Data were collected at the Institute of Development, Aging, and Cancer of Tohoku University. On the day of the visit, the participants underwent brain MRI, global intelligence test, face morphological scanning, and 2D palm scanning. On the same day, they provided saliva samples and answered the questionnaires. Parents brought the mother and child health handbook and the score reports of their offspring’s physical fitness test. Several questionnaires were administered in this study. To reduce the psychological burden on participants as much as possible, most questionnaires were taken home so that they could be self-administered at the participants’ own pace. In addition, a time schedule for participation was documented on the website to help participants keep a track of the progression of tests in the study. Participants were asked to check the time schedule in advance. Questionnaires taken home were requested to be submitted by post within approximately 2 weeks of the date of participation.

The duration of all experiments on the day of participation is approximately 4 h, and the time required to complete the questionnaire test to be taken home is approximately 1 h.

#### 2.4.2. Sociological information

Participants were asked to provide the following information through originally formatted questionnaires: educational background (69), personal annual income, employment status, type of business, job role, changes in income due to the COVID-19 pandemic (70), family members, sibling composition, availability, and type of pets. In the case of the participating offspring as students, questions about occupation and income were omitted.

#### 2.4.3. Health and physical information

Height and weight were measured and recorded before the MRI.

All participants answered the Japanese version of the Flinders handedness questionnaire (FLANDERS) (71, 72) to evaluate handedness.

Participants were asked about their current illness under treatment, medications they are currently taking, medical history, history of COVID-19 infection, and history of psychiatric diseases and developmental disorders in relatives, using the original survey form. For participants aged under 15, questions other than those regarding the history of COVID-19 infection were answered by the mother. Female participants also answered questions on the menstrual cycle and the last menses start date.

Pubertal status was also assessed for underage participants using the Pubertal Development Scale (73, 74), a self-administered questionnaire comprising five questions about the progression of secondary sexual characteristics.

Only parents of children aged under 18 were asked to bring their offspring’s score reports from physical fitness tests conducted recently at school. The Physical Fitness Test is an official physical ability test mandated by the Japan Ministry of Education, Culture, Sports, Science, and Technology to investigate the nation’s physical strength and athletic ability, and is administered annually at schools. This test score captures a child’s flexibility and agility. The score reports were scanned by the research staff.

#### 2.4.4. Global intelligence

To measure global intelligence, participants aged 16 or older completed the WAIS-IV, and those aged 15 or younger completed the WISC-IV. Ten core subtests were conducted to calculate the full-scale intelligence quotient along with four factors (verbal comprehension, perceptual reasoning, working memory, and processing speed indices). The tests were conducted in a quiet, non-stimulating room, one-on-one with staff familiar with the procedure and participants. The time required to complete the test ranged approximately 60–90 min.

#### 2.4.5. Brain imaging

Prior to scanning, participants were asked to confirm that they had no metal in their bodies, tattoos, permanent makeup, claustrophobia, nyctophobia, pregnancy, and were not wearing thermal undershirts and contact lenses. Participants’ signatures were obtained for this confirmation.

Brain images were acquired using a 3-Tesla dStream Achieva scanner (Philips Medical Systems, Best, Netherlands) with a 20-channel headneck coil. Participants wore earplugs and headphones to protect their ears from MRI noise. An emergency buzzer was given to the participants to alert the research staff if any problems occurred during the imaging. The total scan time was approximately 24 min.

The following images were acquired for each participant. First, sagittal T1-weighted images (T1WIs) were obtained using a magnetization-prepared rapid gradient-echo (MPRAGE) sequence. Second, sagittal T2-weighted images (T2WIs) were acquired using a spin-echo sequence. Third, resting-state functional MRI (rsfMRI) was performed using gradient-echo echo-planar imaging (EPI). Each run contained 120 image volumes preceded by 7 dummy volumes, with each volume comprising 45 slices. Participants were asked to stay awake, not think about anything as much as possible, and gaze at the center of the black fixation point on the gantry. Fourth, field maps were acquired for distortion correction using rsfMRI. As the usefulness of each type of distortion correction remains controversial, we obtained two types of field maps: images with a double-echo spoiled gradient echo sequence (GRE-field map) and a pair of spin-echo EPI images with opposite anterior–posterior (AP) and posterior–anterior (PA) phase encoding direction (SE-oppPE-field map) (75–77). Fifth, diffusion-weighted images (DWI) was obtained in 30 different directions using a spin-echo sequence. The *b*-value was set to 1,000s/mm^2^ for 30 volumes. Sixth, two non-diffusion weighted (*b*=0) images were acquired with reversed-phase encoding directions (AP and PA) for distortion correction (78). Details of the acquisition parameters are presented in Table 1.

**Table 1:** Acquisition parameters of brain images.

	TR (ms)	TE (ms)	FOV RL x AP (mm)	Matrix RL x AP	Slices	Slice thickness	Flip angle	Phase encoding direction	Acq time
T1WI	11	5.1	256 × 256	368 × 368	257	0.7	8	LR	5 min 19 s
T2WI	2,500	3,200	256 × 256	368 × 368	250	0.7	90	LR	5 min 55 s
rsfMRI	2,500	30	220 × 220	64 × 64	45	3.4	80	PA	5 min 15 s
GRE-field map	488	4.92/7.38	220 × 220	64 × 64	45	3.4	60	AP	33 s
SE-oppPE field map1	6,100	60	220 × 220	64 × 64	45	3.4	90	AP	12 s
SE-oppPE field map2	6,100	60	220 × 220	64 × 64	45	3.4	90	PA	12 s
DWI	10,000	80	224 × 224	112 × 112	75	2.0	90	AP	5 min 30 s
b0	10,000	80	224 × 224	112 × 112	75	2.0	90	PA	1 min

T1WI, T1-weighted images; T2WI, T2-weighted images; rsfMRI, resting-state functional MRI; GRE-field map, field map with a double-echo spoiled gradient echo sequence; SE-oppPE field map, field map with spin-echo echo planar imaging and opposite phase encoding direction; DWI, diffusion-weighted images; b0, non-diffusion-weighted image with b-value = 0. TR, repetition time; TE, echo time; FOV, field of view; RL, right to left; LR, left to right; AP, anterior to posterior; PA, posterior to anterior; Acq time, acquisition time; ms, milliseconds; mm, millimeters.

#### 2.4.6. Genome and epigenome

Saliva samples were collected for genetic/epigenetic analysis using Oragene^®^ Discover (DNA Genotek, Inc., Ottawa, ON, Canada) following the manufacturer’s protocol. The collected samples were stored at room temperature until DNA extraction was performed. Genomic DNA was extracted from the samples using an Oragene^®^ purifier. Thereafter, the extracted DNA samples were measured for concentration using a Nanodrop 2000 (Thermo Fisher, Applied Biosystems, Foster City, CA, United States) and then stored in a freezer at −30°C. In future research, we plan to use microarrays for single-nucleotide polymorphism (SNP) typing and epigenomic analyses.

#### 2.4.7. Psychological measures

We used psychological questionnaires to collect information about the participants’ behavioral phenotypes, including mental health problems, well-being, personality traits, and socioemotional competencies. We selected scales for which reliability and validity were confirmed and are widely used internationally.

Our first priority was to assess behavioral phenotypes using the same scale for both parents and offspring. However, because it is difficult for children aged under 15 to answer the same questionnaires as adults, we adopted appropriately developed scales for children whenever possible. Moreover, self-administered questionnaires were used unless only parent-administered questionnaires for children were available.

The response burden for questionnaires varies by respondents’ age. Children are more likely to be burdened (79). Therefore, to roughly equalize the response burden, fewer questionnaires were administered to offspring aged 10–15 compared with those aged 16 and older. Which participants answered which questionnaires are summarized in Tables 2–4.

**Table 2:** Questionnaires assessing mental health problems and well-being.

	Trios of offspring aged under 15 and their parents		Trios of offspring aged over 16 and their parents		
Objectives	Offspring	Parents	Offspring	Parents	Number of items, scale types
Depressive symptoms	DSRS-C	K6	K6	K6	DSRS-C: 9 items, 3-point LS K6: 6 items, 5-point LS
Subjective happiness	SLSS	SWLS	SWLS	SWLS	SLSS: 7 items, 6-point LS SWLS: 5 items, 7-point LS
Internalizing/externalizing symptoms	Self-rated SDQ for 11–17 year olds	SDQ for the parents of 4–17 year olds	x	x	25 items, 3-point LS
Trauma and re-experiencing symptoms	x	PDS	PDS	PDS	See main text for details.
Loneliness	x	x	TIL	TIL	3 items, 5-point LS
Fear of COVID-19	FCV-19S	FCV-19S	FCV-19S	FCV-19S	7 items, 5-point LS

“x” means that the questionnaire is not administered to that participant. DSRS-C, Birleson Depression Self-Rating Scale for Children (80, 81); K6, Kessler 6 Psychological Distress Scale (82, 83); SDQ, Strength and Difficulties Questionnaire (84); TIL, Three-Item Loneliness Scale (85, 86); FCV-19S, Fear of COVID-19 Scale (87, 88); LS, Lickert scale.

**Table 3:** Questionnaires assessing personality traits and socioemotional competencies.

	Trios of offspring aged under 15 and their parents		Trios of offspring aged over 16 and their parents		
Objectives	Offspring	Parents	Offspring	Parents	Number of items, scale types
Extraversion, agreeableness, conscientiousness, neuroticism, openness	Big Five (J.H.S.) Big Five-C (E.S.) TIPI	NEO-FFI TIPI	NEO-FFI TIPI	NEO-FFI TIPI	Big Five: 70 items, yes/no Big Five for children: 51 items, yes/no NEO-FFI: 60 items, 5-point LS TIPI: 10 items, 7-point LS
Temperament	BIS/BAS-C	BIS/BAS	BIS/BAS	BIS/BAS	Either: 20 items, 4-point LS
State and trait anxiety	STAI-C	STAI	STAI	STAI	STAI-C: 40 items, 3-point LS STAI: 40 items, 4-point LS
Schizotypal personality	x	SPQ-Brief	SPQ-Brief	SPQ-Brief	SPQ-Brief: 22 items, Yes/No
Psychotic like experience	APSS	x	x	x	7 items, “Yes, definitely”/"Maybe”/"No, Never”
Inattention and impulsivity	ADHD-RS (parents-administered)	ASRS	ASRS	ASRS	ADHD-RS: 18 items, 4-point LS ASRS: 18 items, 5-point LS
Autistic traits	AQ-C (parents-administered)	AQ	AQ	AQ	Either: 50 items, 4-point LS
Borderline personality	x	x	SCID-II	SCID-II	15 items, 5-point LS
Negative feelings in peer relationship	x	x	ECR-RS	ECR-RS	9 items, 7-point LS
Emotion regulation strategies	x	x	CERQ	CERQ	18 items, 5-point LS
Empathy	x	x	IRI	IRI	28 items, 5-point LS
Machiavellianism, narcissism, psychopathy	x	x	SD3	SD3	27 items, 5-point LS

“x” means that the questionnaire is not administered to that participant. Big Five, Big Five Personality Inventory (89), J.H.S., junior high school students; Big Five-C, Big Five for children (90); E.S>, elementary student; TIPI, Ten Item Personality Inventory (91, 92); NEO-FFI, NEO-Five Factor Inventory (93, 94); BIS/BAS, Behavioral Inhibition System/Behavioral Activation System Scale (95, 96); BIS/BAS-C, BIS/BAS for children (97, 98); STAI, State–Trait Anxiety Inventory (99, 100); STAI-C, STAI for children (101, 102); SPQ-Brief, Schizotypal Personality Questionnaire Brief (103, 104); APSS, Adolescent Psychotic-Like Symptom Screener (105, 106); ADHD-RS, Attention-deficit hyperactivity disorder-Rating Scale IV (107, 108); ASRS, ADHD Self-Report Scale (109, 110); AQ, Autism-Spectrum Quotient (111, 112); AQ-C, AQ children’s version (113, 114); SCID-II, The personality questionnaire in The Structured Clinical Interview for DSM-5 personality disorders (115, 116); ECR-RS, Experience in Close Relationships Relationship-Structure (117, 118); CERQ, Cognitive Emotion Regulation Questionnaire (119, 120); IRI, Interpersonal Reactivity Index (121, 122); SD3, Short Dark Triad (123, 124); LS, Lickert scale.

**Table 4:** Questionnaires assessing in-home environmental factors.

	Trios of offspring aged under 15 and their parents		Trios of offspring aged over 16 and their parents		
Objectives	Offspring	Parents	Offspring	Parents	Number of items, scale types
Family functioning	FACES-III	FACES-III	FACES-III	FACES-III	20 items, 5-point LS
Family relationship	ICHIGEKI	ICHIGEKI	ICHIGEKI	ICHIGEKI	See main text for details.
Parenting style	CRPBI (self-administered) CRPBI (parents-administered)	PBI	PBI	PBI	CRPBI: 30 items, 4-point LS PBI: 25 items, 4-point LS
ACEs	reference from the survey by the Cabinet Office in Japan	CTQ Felitti’s scale	CTQ Felitti’s scale	CTQ Felitti’s scale	CTQ: 28 items, 5-point LS others: See main text in details.
Satisfaction with marital relationship	x	QMI	QMI	QMI	6 items, 4-point LS
Marital conflicts	x	MSQ	MSQ	MSQ	10 items with 4-point LS and 4 items with “Yes”/"No”

“x” means that the questionnaire is not administered to that participant. FACES-III, Family Adaptation, and Cohesion Scales (125, 126); ICHIGEKI, Inventory for Character of Intra-Inter Generation in Kinship (127); PBI, Parental Bonding Instrument (128, 129); CRPBI, Children’s Report on Parent Behavior Inventory (130–132); CTQ, Childhood Trauma Questionnaire (133, 134); QMI, Quality Marriage Index (135, 136); MSQ, Multidimensional Stress Questionnaire for Couples (137, 138); LS, Lickert scale.

##### 2.4.7.1. Mental health problems and well-being

Participants’ depressive symptoms, subjective happiness, eudaimonic well-being, internalizing/externalizing symptoms, serious trauma, re-experiencing symptoms, loneliness, and fear of COVID-19 were assessed. The names of the questionnaires, number of items, and responses are summarized in Table 2. Here, we discuss only the details necessary for special mention.

The Satisfaction with Life Scale (SWLS) (139, 140) is used to assess subjective happiness. Participants were asked to respond not only for themselves but also for the two others (e.g., the mother answers about herself, her offspring, and her husband) to examine how the trio perceives each other’s perspectives on life, and whether their self-evaluation matches or diverges from the two others’ evaluations. Prior studies have evaluated others’ assessment of well-being using this scale (141). On the other hand, for offspring aged 15 and younger, the Student’s Life Satisfaction Scale (SLSS) (142, 143) was used to evaluate subjective happiness. Trios of offspring aged under 15 and their parents did not evaluate each other’s well-being.

The Strengths and Difficulties Questionnaire (SDQ) (84) was used to assess internalizing and externalizing symptoms in offspring aged 15 and lower. We administered the self-report version of the SDQ for offspring. Additionally, parents were administered the parent-report version of the SDQ to assess the offspring’s internalizing and externalizing symptoms from multiple perspectives. The Japanese version of this questionnaire was obtained from YouthinMind.^12^

The Posttraumatic Diagnostic Scale (PDS) (144–146) was used to assess the presence or absence of serious trauma and re-experiencing symptoms. Respondents selected what they have experienced thus far from a list of several traumatic events, such as natural disasters and assaults; further, if any of the selected events have bothered them in the past month, they were asked to state when the event began and ended, and the extent to which they re-experience the event.

##### 2.4.7.2. Personality traits and socioemotional competencies

Participants were assessed for the following: extraversion, agreeableness, conscientiousness, neuroticism, openness, temperaments of behavioral inhibition/activation, state and trait anxiety, schizotypal personality, psychotic-like experience, inattention and impulsivity, autistic traits, borderline personality, negative feelings in peer relationships (discomfort in close relationships and abandonment anxiety), emotion regulation strategies, empathic traits, Machiavellianism, narcissism, and psychopathy. The names of the questionnaires, number of items, and choices are summarized in Table 3. Here, we discuss only the details necessary for special mention.

The Ten-Item Personality Inventory (TIPI) (91, 92, 147) comprises 10 items to assess five-factor personality traits. Each participant responded about themselves and the two others (e.g., the offspring answered about themself, their mother, and their father) to examine their perception of each other’s personality, and whether their self-evaluations match or diverge from the others.

The Schizotypal Personality Questionnaire Brief (SPQ-Brief) (103, 104) was used to assess traits believed to have a common biological basis with schizophrenia. For offspring aged under 15, the Adolescent Psychotic-Like Symptom Screener (APSS) (105, 106) was used to assess psychotic-like experiences, which are associated with schizophrenia. The APSS consists of seven items—four taken from the schizophrenia section of the Diagnostic Interview Schedule for Children (148, 149) and three items on visual hallucinations, delusions of control, and grandiosity added by Kelleher et al. (105). The Japanese version of the APSS was obtained from the Japanese paper on which Ando et al. were based.

The personality questionnaire in the Structured Clinical Interview for DSM-5 personality disorders (SCID-II) (115) was used to evaluate the tendency toward borderline personality disorder (BPD). We only used 15 items assessing BPD. Although the original format was to answer “yes” or “no,” a 5-point Likert scale is sometimes used to capture individual differences in more detail (150–152), which we adopted in this study.

Experience in Close Relationships Relationship-Structure (ECR-RS) (117) was used to evaluate discomfort in close relationships and abandonment anxiety. Participants were asked to indicate their thoughts on their relationships with people such as their father, mother, friend, and lover/spouse.

##### 2.4.7.3. Environmental factors during development

The definition of environment is broad and includes all “non-genetic” factors (153). According to a previous study that proposed the concept of exposome (all environmental exposures from the prenatal period through the entire life) (153), environmental factors are divided into three domains: an internal domain, comprising metabolism, inflammation, hormones, and other bodily processes that can be quantified using high-throughput molecular omics technology; a specific external domain, comprising the individual-level psychosocial factors that can be assessed by questionnaires, such as educational achievement, social deprivation, and traumatic experience; and a general external domain, comprising community-level physical factors such as temperature, green space, air pollution, transportation, and population density. The findings of large neuroimaging cohort studies with abundant data belonging to the general external domain, such as CHIMGEN (5) and ABCD study® (154), indicate the importance of physical environmental factors in brain development. In contrast, internal domain data are an advantage of birth cohorts (155). As mentioned in the introduction, the interest of this study was to elucidate the mechanisms of intergenerational transmission of experiences and their influences. Therefore, we focused on the environmental factors that corresponded with the specific external domain during the developmental periods of both parents and offspring. The environmental factors that we focused on can be roughly divided into three subdomains: in-home, out-of-home, and pre/postnatal.

###### 2.4.7.3.1. In-home environment

We assessed family functioning, family relationships, perceived parenting style, ACEs, satisfaction with marital relationships, and marital conflicts. The names of the questionnaires, number of items, and responses are summarized in Table 4.

Family Adaptation and Cohesion Scales (FACES-III) (125, 126) were used to assess two dimensions of family functioning: cohesion (emotional bonding among family members) and adaptability (the ability to change power relations and roles within the family depending on the situation).

The Inventory for Character of Intra-Inter Generation in Kinship (ICHIGEKI) (127) was used to identify how respondents perceived their relationships with the two others in the trio. For example, a father will respond with a score of 1–10 for the magnitude of his emotional bonding and conflicts with his wife and offspring.

The Parental Bonding Instrument (PBI) (128, 129) was used to assess emotional warmth and overprotectiveness received from parents before the age of 16. Participants were asked to answer about both their father and mother. For offspring aged under 15, the Children’s Report on Parent Behavior Inventory (CRPBI-30) (130–132) was used to assess parenting style in terms of emotional acceptance and control. Offspring aged under 15 were asked to answer about both their father and mother. The CRPBI-30 scale allows both children and parents to assess parenting style from each perspective, and thus, parents of offspring aged under 15 are also asked to complete this questionnaire.

ACEs were assessed using several questionnaires. First, the Childhood Trauma Questionnaire (CTQ) (133, 134) was used to assess childhood experiences of physical/emotional/sexual abuse and physical/emotional neglect. Second, based on previous studies, we investigated the presence or absence of parental divorce or separation, domestic violence, criminal behavior in the household, mental illness in the household, death of a caregiver, and poverty before age 18 (23, 156). Although mental illness in the household is also included as an ACE in Felitti’s scale, it was omitted in this study because we already obtained this information in the health and physical domains. For offspring aged under 15, the presence or absence of experiences of physical/emotional abuse and neglect, parental divorce or separation, witnessing domestic violence, alcohol or substance abuse addiction in the household, and mental illness in the household were examined based on a survey of child poverty by the Cabinet Office of Japan.

Quality Marriage Index (QMI) (135, 136) was used to evaluate the parents’ satisfaction with marital relationships through questions about bonding. As this scale is also used to assess the offspring’s perception of the relationship between their parents (157), we also asked the offspring participants to answer questions about the bonding between their father and mother. However, owing to the difficulty of the questions, this scale was not administered to offspring aged under 15.

The Multidimensional Stress Questionnaire for Couples (MSQ) (137, 138) was used to assess marital conflict between parents. The questionnaire comprises 10 items rated on a 4-point Likert scale to indicate the presence or absence of daily conflicts occurring in the last 7 days and 1 year; additionally, 4 items on more serious marital problems (e.g., violence and infidelity) were answered with “yes” or “no.”

Using retrospective questions on their experiences up to adolescence, participants were also asked about the frequency of conversations during mealtimes at home, family members who are home when they return from school, the relationship between the spatial composition of their house and their private room, and their experience of pet ownership and emotional unity with their pets.

###### 2.4.7.3.2. Out-of-home environment

We asked participants the following information about their experiences till high school: friendships, relationships with teachers, meeting others who influenced their lives, bullying victimization/perpetration/bystanders, reading, sports, playing a musical instrument, leadership positions, changing schools, living abroad, excitement about nature and the arts, and enthusiasm for a hobby. They were also asked about the elementary, junior high, and high schools that they attended, including the approximate number of students, whether they took entrance exams, and whether they were coeducational schools. Furthermore, we asked about whether they played outdoors or indoors from preschool to elementary school, when they began using cell phones or smartphones, and whether their parents restricted them from using TV, games, or phones. As there are few reliable and validated internationally used scales for the above information, the questions used in the Japanese literature or the survey forms of Japanese public institutions were used as references.

As Japan is prone to natural disasters, we asked respondents about the locations of homes they had lived in by age 20 to capture their experiences with natural disasters. Respondents were asked to indicate in which prefecture and municipality and the ages at which they lived in each home. This question was answered by the mother for offspring aged 15 or younger.

###### 2.4.7.3.3. Pre-and-postnatal environment

To examine the impact of the pre- and postnatal environments on offspring development and intergenerational transmission, we asked mothers about the following information: experience of fertility treatment and artificial abortion prior to conceiving the offspring participating in this study; presence or absence of any perinatal problems such as breech baby; neonatal asphyxia; obstetric complications; perinatal depression; anticipation of childbirth; duration and method of breastfeeding; social support during and after pregnancy; and the frequency of alcohol, caffeine, raw food, and junk food consumption from when the pregnancy was discovered to delivery and from delivery to weaning. Additionally, both mothers and fathers were asked about their smoking habits before, during, and after pregnancy; whether there were smokers around them during their childhood; and whether their working patterns changed due to marriage and childbirth.

This study was conducted in Sendai City, Miyagi Prefecture, Japan, where many residents experienced the Great East Japan Earthquake. In particular, participants born between 2008 and 2013 may have experienced an earthquake either prenatally or as infants. Therefore, we asked their parents about the damage caused by the Great East Japan Earthquake. Parents were asked about the prefecture in which they lived at the time of the earthquake, the extent of damage to their household, and whether their relatives were affected.

Furthermore, perinatal information was also collected from the Mother and Child Health Handbook, which is issued for women in Japan at the time pregnancy is confirmed and is used during pregnancy and postpartum health checkups. This handbook includes information on the mother’s weight, fundal height, blood pressure, fetal growth at each prenatal checkup, method of delivery, birth weight, infant growth, and developmental milestones up to age 6. We scanned all pages after obtaining the mother’s permission.

##### 2.4.7.4. Lifestyle habits

Participants’ lifestyle habits were also evaluated using questionnaires. For participants aged 16 and older (both parents and offspring), physical activity was assessed using the Global Physical Activity Questionnaire (GPAQ) (158, 159); eating and drinking habits were assessed using the Food Frequency Questionnaire (FFQ) short-form (160). Subjective sleep quality was assessed using the Athens Insomnia Scale (AIS) (161, 162), and participants were asked about their awareness of snoring or sleep apnea. Smartphone use and online game habits were assessed using the Smartphone Addiction Scale Short Version (SAS-SV) (163, 164) and Ten-Item Internet Gaming Disorder Test (IGDT-10, the Japanese version was translated by the Kurihama Medical and Addiction Center) (165). We also assessed bathing habits (166, 167). Smoking habits were assessed using the question items used in the survey by the Ministry of Health, Labour and Welfare of Japan was used. Additionally, the Multidimensional Scale of Perceived Social Support (168, 169) was used to ask about help obtained from daily interpersonal relationships.

For offspring aged under 15, physical activity was assessed using the WHO Health Behavior in School-aged Children (HBSC) (170, 171). Eating habits were assessed using the same scale as adults (FFQ short-form); however, as it is difficult for children to assess their own eating habits (172), we asked one of the parents to complete this scale. Additionally, the offspring were asked if they ate breakfast, lunch, and dinner every day. Subjective sleep quality was assessed using five items in the sleep disturbance factor of the General Health Questionnaire (GHQ-30) (173). Children’s sleep quality was assessed using the GHQ-30 in a previous study (174). Additionally, an isolated question asked the offspring whether they went to bed at the same time each day. The Japanese version of the Korean Scale for Internet Addiction for Adolescents (K-scale) was used to assess Internet-use habits. The Japanese version of the K-scale was translated by the Kurihama Medical and Addiction Center, with permission from the National Information Society Agency. Furthermore, we asked the offspring about the average number of study hours per day.

##### 2.4.7.5. Other measurements of phenotypes

To examine the effects of intergenerational transmission on phenotypes other than brain structure and function or behavioral traits assessed by questionnaires, we obtained the following measurements: face morphology, ratio of second to fourth digit length (2D/4D ratio), and characteristics of the drawn tree. The brain and face have been reported to share several genetic loci (175), and facial asymmetry is associated with broader autistic phenotypes (176, 177). Moreover, 2D/4D ratio is considered to reflect sex steroid hormone exposure during embryonic development (178). Several findings have indicated a sex-specific association between the 2D/4D ratio and brain structure or function (179–181). Additionally, the associations between the 2D/4D ratio and personality traits, such as schizotypal personality (182), neuroticism (183), and emotional stability (184), are shown. The tree-drawing test (Baum test) (185) is a projective personality assessment technique. As the characteristics of drawn trees, such as canopy area and trunk width, have been reported to be significantly associated with schizophrenia (186) and depression (187), the usefulness of this tool to quantitatively assess some facets of psychological traits that cannot be captured with questionnaires has been suggested (188). We collected these data from the parents and offspring using the following procedure.

###### 2.4.7.5.1. Face morphology

Digital facial stereophotogrammetry was used to capture 3D facial surfaces; 3D stereophotogrammetric imaging is a well-established approach for generating dense 3D points that represent the surface geometry of a face using multiple 2D images with overlapping fields of view (189). Facial surfaces were obtained using EinScan Pro HD (SHINING 3D Tech. Co., Ltd., Hangzhou, China). According to a standard facial imaging protocol (189), participants were instructed to close their mouths, relax their faces, and maintain a neutral expression. The participants were also asked to keep their eyes closed. 2D face images were also acquired using iPhone 6 (Apple Inc., Cupertino, CA, United States) for reference.

###### 2.4.7.5.2. 2D/4D ratio

Brother PRIVIO DCP-J962N (Brother Industries, Ltd., Aichi, Japan) was used to acquire 2D palm scanned images. According to a prior study’s protocol (190), both hands are placed flat on the scanner glass, ensuring that they are clearly separated from each other. Next, the scanner cover is closed, following which the scanner generates a PDF file of the image (210 mm × 297 mm, 200 dpi resolution).

###### 2.4.7.5.3. Tree-drawing test

A sheet of A4 paper and a 4B pencil were provided to each participant, and they were instructed to “draw a tree on the drawing paper. The purpose is not to see how good or bad you are at drawing, so please draw freely, with little care.” When the participants finished drawing, they were asked to freely describe on the back of the paper what kind of tree they drew and how they felt after drawing it.

### 2.5. Analysis

Brain images will be preprocessed using the analysis software widely used in this field like FreeSurfer^13^ (191), FMRIB Software Library (FSL)^14^ (192), MRtrix3^15^ (193), and Advanced Normalization Tools (194) (ANTs),^16^ and Analysis of Functional NeuroImages (AFNI)^17^ (195). Structural and functional indices such as gray matter volume, cortical thickness, surface area, local gyrification index, white matter fractional anisotropy, and resting-state functional connectivity will be extracted. Quality controls of preprocessed images will be performed by appropriate scripts and manual editing. All raw and preprocessed images are stored in Brain Imaging Data Structure (BIDS) format for data sharing in the future.

One of the major goals of our study is to understand the putative mechanisms of intergenerational transmission, using brain images as endophenotype. To achieve this objective, we consider the following analyses. First, we plan to determine which characteristics in which brain areas are similar between father and offspring and/or mother and offspring, and whether the similarities can be recognized as the neural basis for the intergenerational transmission of behavioral phenotypes. This theme includes a replication of previous studies of intergenerational neuroimaging. In previous studies, parent–offspring brain similarity has often been described using the correlation of the characteristics of brain region of interest (ROI) between parents and offspring (28, 32, 35, 39, 40). Therefore, we will also examine parent–offspring brain similarity based on correlation analysis. The power analysis using G*Power (196, 197) calculated the required sample size of 134, with a moderate effect size (*r* = 0.30, *α* = 0.05, two-tailed, power = 0.95). Second, we also plan to investigate the association between parental ACEs and offspring’s brain structure or function to present additional knowledge of intergenerational transmission of trauma effect. Linear regression analysis is one of the options for examining these associations (41–44, 198–201). The power analysis using G*Power calculated the required sample size is 138, with a moderate effect size [f2 = 0.15, *α* = 0.05, power = 0.95, number of predictors = 5 (i.e., predictor variables = maternal ACEs, paternal ACEs, interactive term of maternal and paternal ACEs, offspring’s age, and offspring’s sex, dependent variable = ROI value of cortical thickness in offspring)]. Third, we will examine whether the effect of parenting style on offspring’s brain development differs depending on whether there is intergenerational transmission of parenting style. Specifically, we will examine the differences in offspring’s (Generation 3; G3) brain development depending on whether parents’ (Generation 2; G2) parenting style toward their offspring is similar to the parenting style they received from their own caregivers (Generation 1; G1) during their childhood. The parenting style of G2 and G1 are assessed using PBI or CRPBI as mentioned above. According to our preliminary analysis performed using different data from the present study, whether positive parenting style (assessed by the care factor in PBI) transmitted between G1 and G2 was related to G3’s cortical thickness with a moderate effect size [analysis of covariance (ANCOVA) was conducted to compare the four groups: high-care G1 and high-care G2, high-care G1 and low-care G2, low-care G1 and low-care G2, and low-care G1 and high-care G2]. In line with this, the sample size required to obtain a moderate effect size by ANCOVA with four groups was calculated to be 279 [*f* = 0.25, α = 0.05, power = 0.95, number of covariates = 2 (i.e., offspring’s age and gender)]. From the above, approximately 310 trios would be required to meet the largest samples for the currently planned analysis, assuming that 10% of the samples are excluded due to incomplete or missing data. In addition to the above, other analyses deemed necessary will be performed. For each analysis, if there are missing values, the data from that participant will be excluded from the analysis.

In the future, the sample size can be increased further to enable analysis using genetic information. In genetic studies, a family-based design has the advantage that it can eliminate the problems of population stratification (202). The parent–offspring trio is the simplest family-based design. Some previous studies using polygenic transmission disequilibrium tests (pTDT), a recently developed family-based design analysis method (203), have been conducted with <100–200 trios (204–206), while others have been conducted with nearly 3,000 trios (203, 207, 208). In contrast, previous studies on the genetic nurture effect often deal with 1,000–2,000 trios (51). Although the optimal sample size of trio-based genetic analysis is unclear, we hope to expand this study by including more than 1,000 trios in the future.

Although this study is a venturesome project with a different budget and staffing compared to existing large cohorts, we intend to eventually develop this project into a multi-site collaborative study and lead the neuroimaging research of parent–offspring trios.

## 3. Discussion

### 3.1. Current status and future directions

By April 2023, we completed data acquisition from 174 trios. The mean ages of the offspring, mothers, and fathers were 17.55 ± 6.76, 50.18 ± 6.21, and 51.58 ± 7.03, respectively. The numbers of male and female offspring were 85 and 89, respectively. Most participants completed all the surveys, but brain images were partially omitted for some due to unexpected claustrophobia or technical errors of the MRI scanner.

### 3.2. Strengths and limitations

Our study has several strengths. First, we were able to accumulate data on fathers, whereas most previous studies have focused only on mothers and offspring (209). We devised to make it easier for fathers to participate in this study by allowing families to separately schedule their participation in the study and by conducting the survey on weekends. Second, data collection was performed in the same manner for both parents and offspring, especially for trios in which the offspring were aged 16 or older. For trios with offspring aged 15 or younger, we attempted to quantify common phenotypes by using age-appropriate self-administered scales for each parent and offspring. This approach allowed us to examine the parent–offspring similarities and their relationships for each phenotype and intermediate phenotype. Third, behavioral phenotypes and biological markers were collected using various methods, including not only brain images and questionnaires but also peer assessment of personality and well-being, projective techniques, face morphology scans, and palm scans. These diverse data of trios provide a novel perspective on the relationship between the formation of individuality and intergenerational transmission.

This study has some limitations. First, because children aged under 10 were not included in this study, it was not possible to determine when the effects of intergenerational transmission on traits become apparent. This limitation should be addressed through future studies targeting younger children and their parents. Second, as our study used a cross-sectional design, we could not investigate the longitudinal changes in intergenerational transmission effects on the offspring’s lifespan development. Ideally, intergenerational transmission should be investigated when two generations are of the same age, because unapparent traits in youth may appear in middle adulthood (6). However, such an ideal study would take a long time to be realized. We believe that our study adopted the most feasible and optimal design that is currently available. Third, most of the environmental factors were assessed retrospectively; therefore, recall bias may have affected the results. Thus, we tried to address this limitation as much as possible by devising questions that can be answered with a simple choice of answer.

### 3.3. Conclusion

The TRIO study is a novel neuroimaging research project that investigated the association between intergenerational transmission and personality development. Our study provides interdisciplinary insights into how individuals’ lives are involved in the construction of the lives of their descendants in the subsequent generation.

## Ethics statement

This study was conducted in accordance with the principles of the Declaration of Helsinki (210). Approval was obtained from the Institutional Review Board of Tohoku University (Approved No. 2022-1-534). Written informed consent and ascent was obtained from all participants before the study. If a participant was aged under 18, written consent was obtained from their parents.

## Author contributions

YT supervised the project. IM, RY, and YT designed the study. IM and RY collected the data. IM wrote the manuscript, which was reviewed by RY and YT. All authors contributed to the article and approved the submitted version.

## Funding

This work was funded by a Grant-in-Aid for Young Scientists (Grant no. 22K13809) and Grant-in-Aid for Transformative Research Areas (A) (Grant no. 22H05209) from the Ministry of Education, Culture, Sports, and Technology (MEXT) and the Japan Society for the Promotion of Science (JSPS) to IM. This work was also funded by the Program for the Creation of Interdisciplinary Research from the Frontier Research Institute for Interdisciplinary Sciences, Tohoku University to IM. This work was also supported by JST SPRING (Grant No. JPMJSP2114) awarded to RY. This work was also supported by a Grant-in-Aid for Scientific Research (B) (Grant no. 19H04211) from the MEXT to YT. This work was supported by JSPS KAKENHI Grant Number JP22H04926 and Grant-in-Aid for Transformative Research Areas — Platforms for Advanced Technologies and Research Resources “Advanced Bioimaging Support” for the curation and analysis of neuroimaging data.

## Conflict of interest

We used BrainSuite, developed by CogSmart, Inc., as an incentive for the participants. YT was the chief scientific officer of CogSmart, Inc. and has obtained approval from the Conflict of Interest (COI) Management Committee of Tohoku University for his involvement in this study. This study was also supported by the joint research fund of CogSmart, Inc., but these funds have not been used to pay for using BrainSuite.

The remaining authors declare that the research was conducted in the absence of any commercial or financial relationships that could be construed as a potential conflict of interest.

## Publisher’s note

All claims expressed in this article are solely those of the authors and do not necessarily represent those of their affiliated organizations, or those of the publisher, the editors and the reviewers. Any product that may be evaluated in this article, or claim that may be made by its manufacturer, is not guaranteed or endorsed by the publisher.

## Footnotes

^1^ https://abcdstudy.org/

^2^ https://imagen-project.org/cgi-sys/suspendedpage.cgi

^3^ https://www.med.upenn.edu/bbl/philadelphianeurodevelopmentalcohort.html

^4^ https://generationr.nl

^5^ http://chimgen.tmu.edu.cn/en

^6^ https://www.fhi.no/en/studies/moba/

^7^ http://www.bris.ac.uk/alspac/

^8^ https://www.developingconnectome.org/

^9^ https://www.megabank.tohoku.ac.jp/english/research/cohortbiobank/birthree/

^10^ https://www.lifelines.nl/

^11^ http://ttcp.umin.jp/index.html

^12^ https://sdqinfo.org/py/sdqinfo/b3.py?language=Japanese

^13^ https://surfer.nmr.mgh.harvard.edu/

^14^ https://fsl.fmrib.ox.ac.uk/fsl/fslwiki/FSL

^15^ https://www.mrtrix.org/

^16^ http://stnava.github.io/ANTs/

^17^ https://afni.nimh.nih.gov/
